# Physiological and Proteomic Adaptation of the Alpine Grass *Stipa purpurea* to a Drought Gradient

**DOI:** 10.1371/journal.pone.0117475

**Published:** 2015-02-03

**Authors:** Yunqiang Yang, Chao Dong, Shihai Yang, Xiong Li, Xudong Sun, Yongping Yang

**Affiliations:** 1 Key Laboratory for Plant Diversity and Biogeography of East Asia, Kunming Institute of Botany, Chinese Academy of Sciences, Kunming, China; 2 Plant Germplasm and Genomics Center, Kunming Institute of Botany, Chinese Academy of Sciences, Kunming, China; 3 Institute of Tibetan Plateau Research at Kunming, Kunming Institute of Botany, Chinese Academy of Sciences, Kunming, China; 4 Key Laboratory of Tibetan Environment Changes and Land Surface Processes, Institute of Tibetan Plateau Research, Chinese Academy of Sciences, Beijing, China; 5 University of Chinese Academy of Sciences, Beijing, China; Northeast Forestry University, CHINA

## Abstract

*Stipa purpurea*, an endemic forage species on the Tibetan Plateau, is highly resistant to cold and drought, but the mechanisms underlying its responses to drought stress remain elusive. An understanding of such mechanisms may be useful for developing cultivars that are adaptable to water deficit. In this study, we analyzed the physiological and proteomic responses of *S. purpurea* under increasing drought stress. Seedlings of *S. purpurea* were subjected to a drought gradient in a controlled experiment, and proteins showing changes in abundance under these conditions were identified by two-dimensional electrophoresis followed by mass spectrometry analysis. A western blotting analysis was conducted to confirm the increased abundance of a heat-shock protein, NCED2, and a dehydrin in *S. purpurea* seedlings under drought conditions. We detected carbonylated proteins to identify oxidation-sensitive proteins in *S. purpurea* seedlings, and found that ribulose-1, 5-bisphosphate carboxylase oxygenase (RuBisCO) was one of the oxidation-sensitive proteins under drought. Together, these results indicated drought stress might inhibit photosynthesis in *S. purpurea* by oxidizing RuBisCO, but the plants were able to maintain photosynthetic efficiency by a compensatory upregulation of unoxidized RuBisCO and other photosynthesis-related proteins. Further analyses confirmed that increased abundance of antioxidant enzymes could balance the redox status of the plants to mitigate drought-induced oxidative damage.

## Introduction

Global warming during the last 100 years has caused substantial changes in temporal and spatial precipitation patterns in many parts of the world [1]. The Tibetan Plateau, sometimes called “the roof of the world”, is an important region for studying plants’ responses to such changes. This is partly because of its extreme altitude (average elevation, >4000 m), and partly because annual precipitation gradually declines from approximately 700 mm to 50 mm from the southeast to the northwest across the Plateau, with accompanying changes in ecosystem types from alpine marsh, grassland, or coniferous forest to alpine or polar desert [2]. Tree-line data indicate that extreme drought conditions have progressed into the northwest part of the Tibetan Plateau over the past 300 years [3]. With increasing aridity, water has become an even scarcer commodity in this region.

Numerous previous studies have shown that plant growth is positively correlated with precipitation gradients, and is primarily limited by drought when annual precipitation is less than ca. 600 mm in the absence of compensating groundwater supplies [4]. Drought conditions induce various adaptive responses in plants. Osmotic adjustment mediated by the production of compatible solutes is a key adaptive response to drought stress in many plants. The accumulation of compatible solutes provides resistance to drought stress, within species-specific limits, by maintaining turgor and cell volume [5]. Thus, analyses of the responses of plants that grow in alpine or polar deserts can improve our understanding of the mechanisms underlying adaptation to extreme drought conditions.

Plants have evolved various survival mechanisms to enhance their drought tolerance, including stress signal perception and transduction and their associated molecular regulatory networks. Reactive oxygen species (ROS) function as signaling molecules to regulate many biological processes in plants [6]. However, accumulation of excess ROS can harm plant cells, because they disrupt metabolic processes and damage cellular components. To avoid oxidative damage, plants have evolved a system of ROS-scavenging enzymes including superoxide dismutase (SOD), ascorbate peroxidase (APX), catalase (CAT), glutathione peroxidase, and peroxiredoxin. Together, these enzymes constitute a highly efficient system to remove ROS and protect plant cells. The plant hormone abscisic acid (ABA), which is synthesized by 9-cis-epoxycarotenoid dioxygenase (NECD), modulates plant tolerance to drought stress [7]. Accumulation of ABA in the leaf can induce stomatal closure and reduce leaf expansion during the early stages of soil drying [8]. Increased concentrations of ABA may significantly decrease the transpiration rate to prevent dehydration of leaf tissues and enhance drought tolerance under prolonged drought stress [6, 7].

Proteomic analyses provide powerful tools to identify proteins in particular samples, to elucidate additional components of biochemical pathway(s), and to analyze post-translational modifications on a small or large scale. Comparative proteomics analyses have been used to explore the relationships between changes in protein abundance and plant stress acclimation. The main methods used in such analyses are two-dimensional electrophoresis (2-DE) to separate proteins, followed by mass spectrometry (MS) analysis for protein identification, although MS is sometimes used for both identification and quantification of proteins [9]. Such analyses can identify certain proteins that differ between tolerant and sensitive genotypes, or characterize the responses of tolerant vs. sensitive species to a given stress factor. Comparative proteomic analyses have revealed some of the proteins that participate in the drought response in several plants. For example, there were increased abundances of ribulose-1,5-bisphosphate carboxylase oxygenase (RuBisCO) activase and Calvin cycle enzymes in drought-treated *Populus euramericana* [10], 14–3–3-like protein, protein kinase 2, serine-threonine kinase, and a hybrid-type histidine kinase in drought-acclimated *Cicer arietinum* [11], and peroxidases in drought-stressed maize roots [12]. Comparative proteomic analyses can increase our understanding of plant stress acclimation and stress tolerance acquisition by providing a detailed picture of functional proteins under stress conditions. Some strategies of drought tolerance have been revealed by proteomic analyses of several non-crop model plants, including *Agrostis*, Norway spruce and *Carissa spinarum* [13–15].

*Stipa purpurea*, a member of the Poaceae family, is a constructive perennial grass species that grows on the alpine steppe and in meadow environments of the Qinghai–Xizang Plateau, the Pamirs Plateau, and high mountains in central Asia. It is a forage species that feeds grassland animals, and it plays important roles in conserving and preserving soil and water, as a windbreak, and in stabilizing sandy soils because of its extensive distribution [16]. On the Qinghai–Xizang Plateau, it is the dominant species in alpine arid (northwest) and semi-arid (midlands) meadows, and it helps to stabilize diverse landscapes because of its outstanding tolerance to harsh environments, including cold, drought, and gale conditions. However, little is known about the adaptive mechanisms that confer its resistance to the harsh environment on the Tibetan Plateau. Because drought is one the main stress factors limiting plant growth on the Tibetan Plateau, we examined the proteomic profile of *S*. *purpurea* to identify the key intrinsic response to drought stress under laboratory conditions. We also detected carbonylated (oxidized) proteins, and quantified the activities of selected proteins that were differentially regulated in plants under drought stress. These results help us to better understand the adaptation of *S*. *purpurea* to drought conditions on the Tibetan Plateau.

## Materials and Methods

### Ethics statement

This field study was permitted by the meadow managers at the Institute of Tibetan Plateau Research, Chinese Academy of Sciences (CAS) and the Grassland Station of Tibet Province. The work was conducted with the approval of, and in cooperation with, the Grassland Station of Tibet Province and local herders. We confirm that the field studies did not involve endangered or protected species. There are no conflicts of ethics or interest to declare.

### Sample collection and drought treatments

Seeds of *S*. *purpurea* were collected from the Tibetan Plateau, China (35° 14′ 37″ N, 98° 51′ 19″ E) for these experiments. The seeds were brought to the laboratory, cleaned and surface-sterilized in a solution of 2% sodium hypochlorite for 15 min, rinsed five times in sterilized water, and then placed in plastic trays lined with wet paper towels. The seeds were left to germinate for 36 h in the dark at 23°C. The seedlings were grown individually in soil pots under controlled conditions (25°C day/23°C night cycles; 200 mmol photons m^−2^s^−1^ light intensity; relative humidity 75%–80%). When the third leaf was fully expanded, a continuous drought treatment was applied by withholding watering. Samples of *S*. *purpurea* were harvested at 0 d (as control), 1 d, 3 d, 7 d, and 14 d of the drought treatment, immediately frozen in liquid nitrogen, and kept at −80°C until protein and RNA isolation was performed. Three replicates per treatment were collected and analyzed. Soil samples were collected at each sampling time. The soil fresh weight (FW) was measured, then dry weight (DW) was determined after drying soil samples at 105°C for 48 h. Relative soil water content (RWC) was calculated using the formula RWC = [(FW−DW)/DW] × 100%.

### Physiological analyses

Chlorophyll fluorescence was measured using Pulse Amplitude Modulation (PAM) Chlorophyll Fluorometer. To measure the maximum quantum yield of photosystem II (PS II), plants were dark-adapted for 30 min. The Fv/Fm value was recorded during a saturating photon pulse (4000 μmol m^−2^ s^−1^) using a whole plant. Leaf stomatal conductance was measured using a LICOR 6400 portable gas analyzer with a light-emitting diode light source during the drought stress treatment. The RWC of *S*. *purpurea* seedlings was measured according to Yamasaki *et al*. with slight modifications [17]. To estimate FW, each sample was weighed immediately after collection. To obtain turgid mass (TM), each sample was floated in distilled water in a closed petri dish, and then weighed when its moisture content was saturated. The samples were then desiccated using silica in a sealed box and finally oven-dried for 24 h at 80°C to a constant DW. The RWC values were calculated as follows: RWC (%) = [(FW−DW)/ (TM−DW)] × 100.

The malondialdehyde (MDA) content in leaf tissues was measured as described by Duan [18]. Fresh leaves (approximately 0.5 g) were homogenized in 10 mL 10% (w/v) trichloroacetic acid (TCA) and then centrifuged at 12 000 × *g* for 10 min. Then, 2 mL 0.6% (w/v) thiobarbituric acid in 10% TCA was added to 2 mL of the supernatant. The mixture was heated in boiling water for 30 min and then quickly cooled in an ice bath. After centrifugation at 10 000 × *g* for 10 min, the absorbance of the supernatant was measured at 450, 532, and 600 nm.

### Protein extraction

Each plant sample (1 g) was ground in liquid nitrogen using a mortar and pestle, and then total soluble proteins were extracted at 4°C in 5 mL 50 mM Tris-HCl buffer (pH 7.5) containing 20 mM KCl, 13 mM dithiothreitol (DTT), 2% (v/v) NP-40, 150 mM PMSF, and 1% (w/v) PVPP. The homogenate was centrifuged at 12 000 × *g* for 15 min at 4°C, then five volumes of acetone containing 10% TCA and 1% DTT was added to the supernatant. The samples were kept at −20°C for 4 h and then centrifuged at 25 000 × *g* for 30 min at 4°C. The resulting pellet was washed with acetone containing 1% (w/v) DTT, incubated at −20°C for 1 h, re-centrifuged, and then resuspended in acetone. After another centrifugation step, the pellet was vacuum-dried and then dissolved in urea buffer [8 M urea, 20 mM DTT, 4% (w/v) 3-[(3-cholamidopropyl) dimethylammonio]-1-propane-sulfonate (CHAPS), and 2% (v/v) ampholyte (pH 4–7)]. The solution was vigorously mixed with a vortex mixer for 1 h at room temperature, centrifuged at 20°C for 20 min at 25 000 × *g*, and then the supernatant was collected. The final supernatant was used as the total soluble protein extract. The protein concentration in extracts was measured using the Bradford method [19] with bovine serum albumin as the calibration standard. Samples were used immediately or stored at −80°C until analysis.

### Two-dimensional electrophoresis and image analysis

For 2-DE, first-dimension electrophoresis was performed using immobilized pH gradient (IPG) strips (pH 4–7, nonlinear, 17-cm). The IPG strips were rehydrated overnight in 340 μL rehydration buffer [8 M urea; 4% (w/v) CHAPS; 20 mM DTT; 2% (w/v) IPG buffer, pH 3–10 or 4–7; bromophenol blue]. Each strip was loaded with 900 μg protein. The proteins were then subjected to isoelectric focusing at 20°C under the following conditions: active rehydration at 50 V for 14 h, a linear increase to 200 V over 1 h, 500 V for 1 h, a linear increase to 1000 V over 1 h, 8000 V for 2 h, 8000 V to 60 000 V·h, and held at 500 V for 1 h. After isoelectric focusing, the IPG strips were equilibrated for 20 min in equilibration buffer [6 M urea, 20% (w/v) glycerol, 2% (w/v) sodium dodecyl sulfate (SDS), and 50 mM Tris-HCl, pH 8.8, containing 1% (w/v) DTT], and then alkylated with 2.5% (w/v) iodoacetamide in equilibration buffer for 20 min. The strips were transferred to 12% SDS-PAGE gels with no stacking gel. The proteins were then electrophoretically separated at 15°C and 3 W/gel for 1 h and then 15 W/gel. The 2-DE micropreparative gels (triplicates per experimental treatment) were stained with Coomassie Brilliant Blue R-250, then scanned using a GS-800 calibrated densitometer and analyzed with PDQuest software. The optimized parameters were as follows: partial threshold, 4; saliency, 2.0; minimum area, 50. To verify the autodetected results, all spots were manually quantified by determining the ratio of the volume of each detected spot to the total volume of all spots on the gels.

### Protein identification and database searching

Protein spots were manually excised, washed three times with Milli-Q water, and then digested in-gel as described by Wang et al., with minor modifications [20]. Gel slices were reduced with 30% (v/v) acetonitrile in 50 mM ammonium bicarbonate before DTT/iodoacetamide alkylation. The proteins in the gel were digested with trypsin at 37°C for 16 h. The peptides were extracted three times with 0.1% (v/v) trifluoroacetic acid and 50% (v/v) acetonitrile, then MS analysis was conducted using a MALDI-TOF/TOF mass spectrometer 4800-plus Proteomics Analyzer. Primary and secondary mass spectra were transferred to Excel files and submitted to Mascot (http://www.matrixscience.com) for protein identification, applying the following parameters: no molecular weight restriction, one missed trypsin cleavage allowed, iodoacetamide-treated cysteine, oxidation of methionine, a peptide tolerance of 100 ppm, and an MS/MS tolerance of 0.25 Da. Protein identifications were validated manually with at least three peptides matched. Keratin contamination was removed, and the MOWSE threshold was set over 60 (*p* < 0.05). In the Mascot probability analysis, only significant hits were accepted for the identification of a protein sample.

### Analysis of antioxidant enzymes

The activities of APX and SOD were determined using methods described elsewhere [21, 22]. Briefly, to measure SOD activity, each 3 mL reaction mixture consisted of 50 mM sodium phosphate buffer (pH 7.8), 13 mM methionine, 75 μM nitroblue tetrazolium, 16.7 μM riboflavin, and an appropriate volume of enzyme extract. The reaction was initiated by light illumination. One unit of SOD activity was defined as the amount of enzyme that causes a 50% decrease in the inhibition nitroblue tetrazolium reduction under these conditions. The activity of APX was determined by following the decrease in absorbance at 290 nm. The reaction mixture contained 50 mM sodium phosphate buffer (pH 7.0), 1 mM ascorbate, 2.5 mM H_2_O_2_, and an appropriate volume of enzyme extract.

### In-gel enzyme activity staining

Equal amounts of protein from each sample were loaded onto discontinuous PAGE gels, separated under nondenaturing conditions (4°C, 4 h, 35 mA), and then further separated for native-PAGE activity staining using 10% (w/v) acrylamide slab gels as described by Davis [23]. The activities of SOD and CAT were then determined by in-gel staining as described by Greneche [24].

### Detection of hydrogen peroxide

The formation of H_2_O_2_ was visualized as fluorescence of 2′, 7′-dichlorodihydrofluorescein diacetate (H_2_DCF-DA) infiltrated into the root regions as described by Behl [25]. Briefly, *S*. *purpurea* roots were washed twice to remove traces of the treatment agents, and then incubated with H_2_DCF-DA (15 μM) for 30 min. A laser-scanning confocal microscope was used to observe fluorescence (excitation at 488 nm, emission at 515 nm).

### Detection of carbonylated proteins

Carbonylated proteins were analyzed according to Job [26] with slight modifications, as follows. The protein extraction buffer [50 mM Tris-HCl, 20 mM KCl, 13 mM DTT, 2% (v/v) NP-40, 150 mM PMSF and 1% (w/v) PVPP, pH 7.5] contained SDS at final concentration of 0.8% (w/v). Following dialysis, four volumes 10 mM DNPH/2 M HCl was added to the supernatant. The samples were vibrated for 30 min at room temperature, five volumes of 20/80 ice-cold TCA–acetone containing 1 mM DTT was added, and then the mixture was centrifuged at 25 000 × *g* for 30 min at 4°C. The resulting pellet was washed three times with 1 mL 1:1 (v/v) ethanol: ethyl acetate, and then incubated at −20°C for 1 h. After a further centrifugation step, the pellet was vacuum-dried and then dissolved in urea buffer [8 M urea, 20 mM DTT, 4% (w/v) CHAPS, and 2% (w/v) ampholyte pH 4–7]. The *S*. *purpurea* protein samples were separated by 1D- or 2D-PAGE using 12% (w/v) polyacrylamide slab gels and protein identification and database searching were conducted as described above. Carbonylated proteins were detected by western blotting using the anti-DNP immunoassay, as described below.

### Measurement of ABA, glycine betaine, and soluble sugars contents, and RuBisCO activity

Abscisic acid (ABA) was quantified as described previously [27]. Samples were extracted overnight in 1 ml ABA extraction buffer (methanol, containing 100 mg/L butylated hydroxyl toluene, 500 mg/L citric acid monohydrate) according to the instructions of the Phytodetek competitive ELISA kit. Samples were then centrifuged 25 000 × g for 30 min at 4°C. The supernatant was dried and re-suspended in 100 μL 100% (w/v) methanol, and then 900 μL 1 × TRIS-TBS buffer was added. Then, ABA was quantified using the Phytodetek kit, according to the manufacturer’s instructions.

The glycine betaine concentration in the samples was determined according to Costa *et al*. [39]. Briefly, 0.5 g dry plant material was homogenized in 2 mL distilled water and mechanically shaken for 4 h at 25°C. The sample was then centrifuged, and 250 μL of supernatant was mixed with an equal volume of 2 mol/L H_2_SO_4_ and incubated in ice-cold water for 1 h. Cold potassium iodine-iodine reagent (200 μL) was added, and the mixture was gently mixed by vortexing. The samples were incubated at 4°C for 16 h, and then centrifuged at 10 000 × *g* for 15 min at 4°C. The supernatant was carefully aspirated with a 1-mL micropipette, and the pellet was dissolved in 3 mL 1,2-dichloroethane. After 2–3 h, the absorbance was measured at 365 nm with an ultraviolet–visible spectrophotometer. Glycine betaine reference standards were prepared in 2 mol/L sulfuric acid according to Costa [28].

The concentrations of soluble sugars in the samples were determined following Kochert [29]. The activity of RuBisCO was determined by enzymatically coupling ribulose-1,5-bisphosphate carboxylation to nicotinamide-adenine dinucleotide oxidation, which was monitored at 340 nm as described by Xu [30].

### Western blotting analysis

For western blotting analyses, proteins were electrotransferred to polyvinylidene difluoride membranes using a Trans-Blot SD instrument. After transfer, the membranes were blocked with 5% (w/v) powdered milk solution for 1 h at room temperature (23–25°C) and then incubated with the primary antibody, which was diluted to 1:3000 (anti-DNP), 1:1000 (anti-ABA), 1:3000 (anti-dehydrin), 1:3000 (anti-HSP) and 1:3000 (anti-Actin), at room temperature for 1 h. Then, the membranes were incubated with horseradish peroxidase-conjugated secondary antibody for 1 h at room temperature. Antibodies were obtained from Agrisera, and chemiluminescence signals were detected using an ECL kit.

## Results

### Physiological responses of *S*. *purpurea* seedlings to drought stress

**Figure 1:**
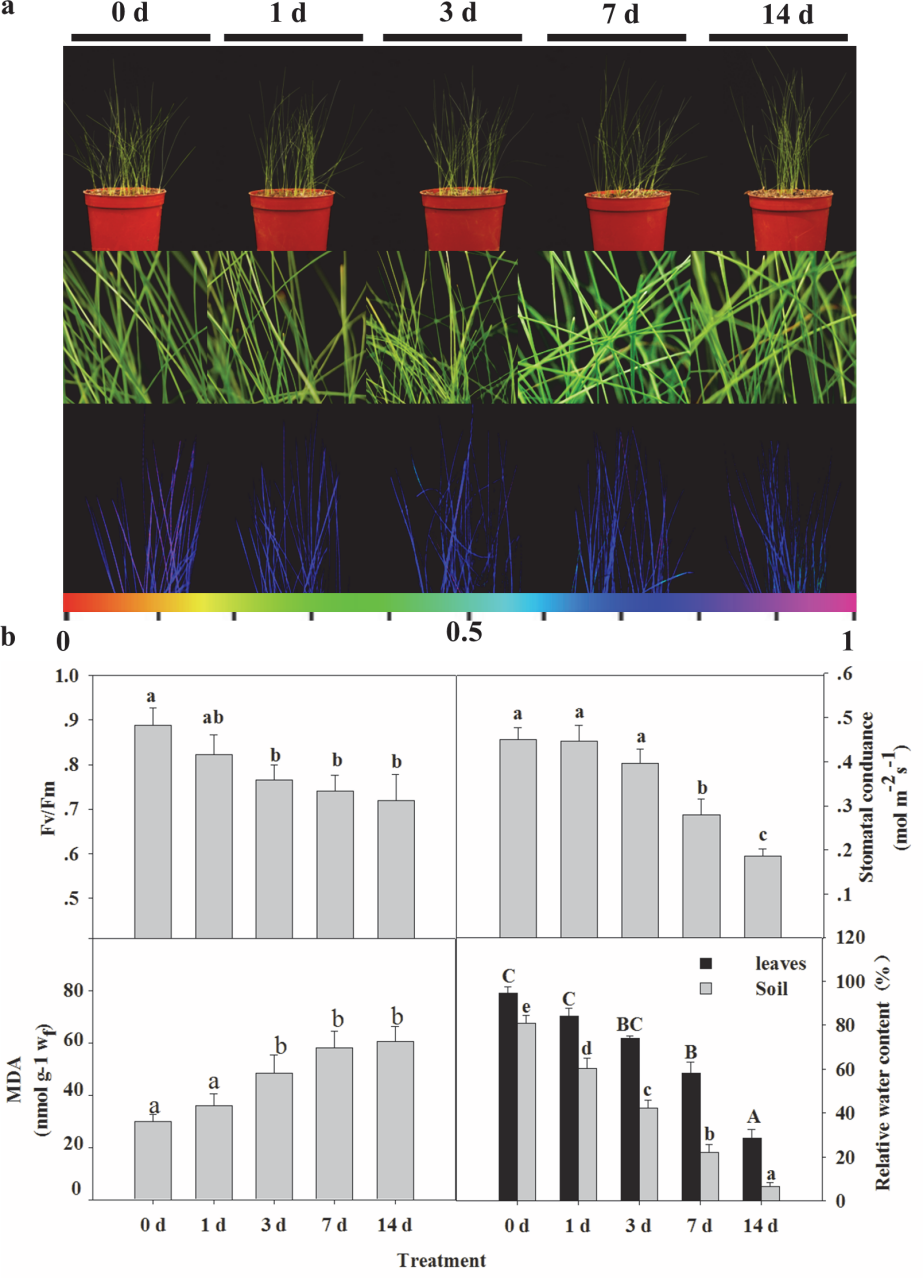
Effects of drought stress on growth of *S*. *purpurea* seedlings. (A) Leaf phenotypes of *S*. *purpurea* following exposure to drought stress. Images were acquired at indicated times during the drought treatment (top); enlarged images (middle); photosynthetic capabilities recorded by Fv/Fm imaging using a PAM chlorophyll fluorometer (bottom). Pseudocolor code depicted at the bottom of the images ranges from 0 (red) to 1.0 (purple). (B) Effects of drought stress on photosynthetic Fv/Fm ratio, stomatal conductance, and MDA content of *S*. *purpurea* seedlings. Relative water content (RWC) of soil and *S*. *purpurea* seedlings were measured as described in “Materials and Methods”. Data are mean values ± SE obtained from three independent experiments (*n* = 15). Different letters within a column indicate significant differences (*p*<0.05; one-way analysis of variance and Tukey’s test).

To analyze the physiological responses of *S*. *purpurea* to drought, we determined the water content of leaves under a drought gradient. The leaves of *S*. *purpurea* remained greenish and vigorous even when the RWC decreased to approximately 5% (Fig. 1A and B). Stomatal conductance determines the speed at which water evaporates through the stomata of plants, and is directly related to the size of the stomatal aperture. Leaf stomatal conductance did not change significantly after 1 d of drought treatment, but decreased rapidly from 3 d to 14 d of drought treatment (Fig. 1B).

Next, we compared the maximum photochemical efficiency of PS II in the dark-adapted state (Fv/Fm), which reflects the photosynthetic efficiency of the plant. The PS II maximum efficiency, as reflected by Fv/Fm values, was lower in drought-treated plants than in controls, but did not decrease significantly in drought-treated plants from 3 d to 14 d of drought treatment (Fig. 1B) (*p*<0.05). The concentration of MDA is a widely used marker of the degree of oxidative damage to plasma membrane lipids. Drought caused a significant change in MDA concentrations in *S*. *purpurea* leaves. The leaf MDA concentration increased to only twice its minimum value by the end of the drought treatment. These results suggest that the drought treatment enhanced the potential of *S*. *purpurea* to tolerate further drought.

### Protein profiling of *S*. *purpurea* seedlings across drought gradients

**Figure 2:**
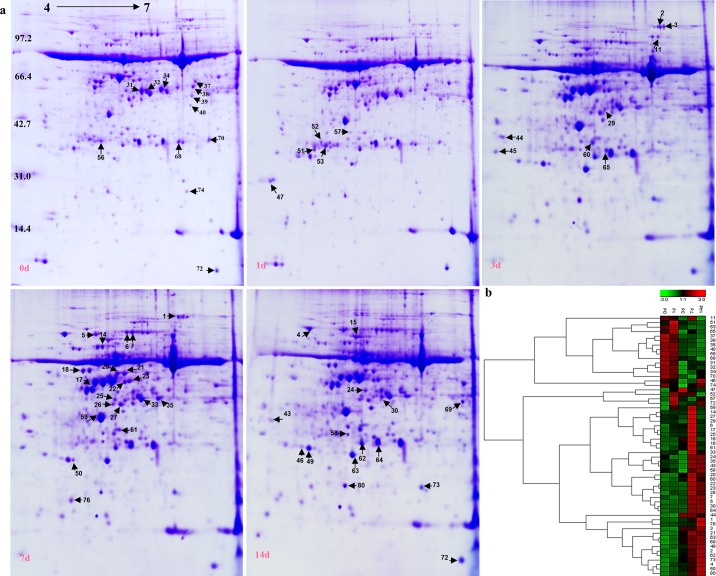
Two-dimensional eletrophoresis maps showing protein profiles of *S*. *purpurea* seedlings under a drought treatment for 0, 1, 3, 7 and 14 days. (A). Proteins were extracted from leaves and separated by isoelectric focusing on an IPG strip (pH 4–7, from left to right), followed by 12% SDS-PAGE. Proteins were visualized by CBB R-250 staining. Arrows indicate proteins showing significant changes in abundance under drought. (B) Hierarchical cluster analysis based on protein expression levels was performed with Genesis 1.7 software. Colors correspond to log-transformed values of protein fold-change ratios (shown in bar at top of figure).

**Figure 3:**
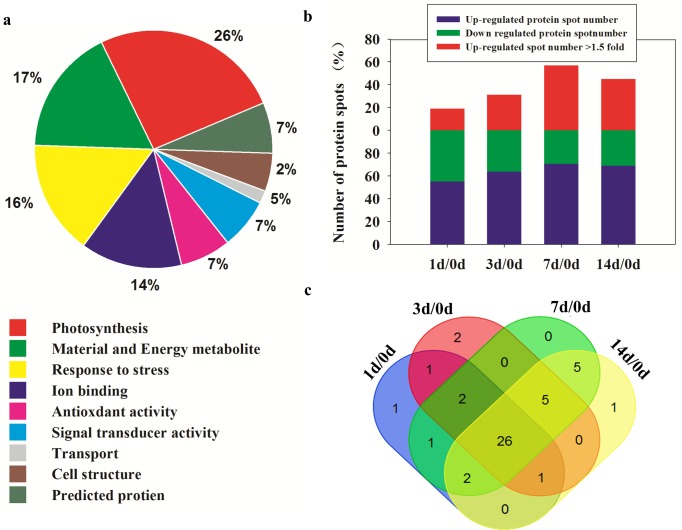
Differentially expressed proteins in *S*. *purpurea* seedlings under drought. (A). Functional classification of differentially expressed proteins in *S*. *purpurea* seedlings under drought. (B) Number of identified proteins showing changes in expression in each treatment (C). Venn-diagram showing overlap of upregulated proteins among 1d/0d, 3d/0d, 7d/0d, and 14d/0d.

To evaluate the effects of drought on the proteomic profiles of *S*. *purpurea* seedlings, total proteins were extracted from leaves of seedlings at various RWCs and then separated by 2-DE (Fig. 2A). We detected 58 differentially expressed proteins (at least 1.5-fold difference between control and drought-stressed plants, *p*<0.05). These proteins were detected and analyzed using PDQuest 7.1 and Genesis 1.7 software (Fig. 2B) [31], and by MALDI-TOF/TOF MS analysis. The identified proteins were assigned Gene Ontology (GO) annotations by comparing their peptide sequences with those in NCBInr databases using the Mascot search engine and Blast2GO software [32]. The proteins and their putative molecular functions are shown in Table 1. The 58 proteins were classified into nine functional categories: photosynthesis (26%), material and energy metabolite (17%), response to stress (16%), ion binding (14%), antioxidant activity (7%), signal transducer activity (7%), transport (5%), cell structure (2%), and predicted protein (7%) (represented by proteins detected in 17, 5, 2, 3, 8 and 6 spots, respectively) (Fig. 3A). The abundance of these proteins changed to different extents during the drought treatment (Fig. 3B). The number of down-regulated proteins decreased during the drought treatment, while the number of upregulated proteins increased, especially those that were upregulated by more than 1.5-fold (Fig. 3B). Thirty proteins were identified as upregulated in the following comparisons: 1 d/0 d, 3 d/0 d, 7 d/0 d, and 14 d/0 d (Fig. 3C). These results indicated that the processes involving these proteins were important for drought adaptation in *S*. *purpurea* seedlings.

**Table 1:** Details of differentially expressed proteins in *S*. *purpurea* leaves.

Spot	NCBI Accession	Protein name	Exp.	Theo.	Score^d^	SC^e^	p-	Sequence	Ratio			
NO.	NO.^a^		Mw/pI^b^	Mw/pI^c^			value^f^		1d/0d	3d/0d	7d/0d	14d/0d
Photosynthesis												
14	gi|2493650	RuBisCO large subunit-binding protein subunit beta	88.9/5.18	53.7/4.88	66	9.42	0.04	ENIGAKL	1.22	1.23	4.39	0.93
								VIAAGANPVQITR				
								NARDLINVLEEAIR				
								GTAAKVVLTKEST				
16	gi|10720253	Ribulose bisphosphate carboxylase/oxygenase activase B	66.4/5.19	47.4/7.59	122	14.94	0.01	KVKNYYH	1.68	11.47	25.6	1.41
								ITRGKGIVD				
								YQPPIMPIGR				
								VPIIVTGNDFSTLYAPLIR				
								EGPPTFDQPKMTIEKL				
17	gi|125580	Phosphoribulokinase	62.8/5.04	45.5/5.72	121	12.38	0.01	NSGFRQN	1.62	1.67	4.17	1.44
								KANDFDLMYE				
								QRDMAERGHS				
								YFGQEVSVLEM				
								IRDLYEQI IAER				
23	gi|108705994	Glyceraldehyde-3-phosphate dehydrogenase B	64.1/5.57	34.0/4.99	94	11.01	0.02	GKLIKVVS	1.16	1.04	3.74	3.13
								TGITADDVNAAFR				
								VVAWYDNEWGYSQR				
31	gi|109940135	Ribulose bisphosphate carboxylase/oxygenase activase	57.9/5.6	51.8/5.43	146	10.73	0.01	KLKKQV	0.78	0.18	0.52	0.52
								FVDSLFQA				
								VPIIVTGNDFSTLYAPLIR				
								IVDSFPGQSIDFFGALR				
32	gi|109940135	Ribulose bisphosphate carboxylase/oxygenase activase	57.7/5.67	51.8/5.43	75	12.45	0.02	KKLKKQ	0.92	0.41	0.67	0.61
								DQQDITRG				
								AKMGINPIMM				
								VPIIVTGNDFSTLYAPL				
								IVDSFPGQSIDFFGALR				
33	gi|218155	Chloroplastic aldolase	56.3/5.71	42.4/7.60	78	11.08	0.04	QAAAPKPV	1.01	0.92	1.2	1.07
								LASIGLENTEANR				
								EAAYYQQGAR				
								WHVSFSYARALQ				
34	gi|109940135	Ribulose bisphosphate carboxylase/oxygenase activase	58.6/5.87	51.8/5.43	156	9.66	0.03	NYHGKSS	0.88	0.16	0.39	0.63
								DQQDITRG				
								VPIIVTGNDFSTL				
								IVDSFPGQSIDFFGALR				
35	gi|8272480	Fructose 1,6-bisphosphate aldolase precursor	57.5/5.95	42.1/9.01	66	11.6	0.02	KKSEWG	0.95	0.74	1.49	1.43
								LASIGLENTEA				
								EAAYYQQGARFAK				
								NAMNQAPNPWHVSFS				
37	gi|357123886	Fructose-bisphosphate aldolase	63.3/6.33	38.0/6.86	134	11.39	0.02	PGKGILA	0.87	0.66	0.68	0.63
								GLDSLGARC				
								VGAEVIAEYTVA				
								ENVAAAQATFLAR				
39	gi|300709536	Regulatory protein Crp	55.0/6.29	28.2/4.26	124	12.31	0.01	KRDAT	0.84	0.6	0.57	0.44
								VSKEGVD				
								AIEEDQSVT				
								VTVVSLPRVHEG				
68	gi|357130587	Carbonic anhydrase	35.8/6.11	51.3/8.90	105	7.17	0.03	TVAPRA	0.93	0.77	0.74	0.57
								EPLKAGQA				
								RVCPSVTLG				
								LEPGEAFTVR				
70	gi|21684929	Ribulose-1,5-bisphosphate carboxylase/oxygenase large subunit	36.6/6.52	51.0/6.60	219	8.72	0.02	GGLDFTKDD	0.87	0.57	0.44	0.72
								ENVNSQPFMR				
								DNGLLLHIHR				
								EMTXGFVDLLR				
73	gi|357163943	Glyceraldehyde-3-phosphate dehydrogenase A	23.7/6.38	43.1/7.01	68	12.41	0.01	NATSED	1.4	2.02	2.42	3.27
								INGFGRIGR				
								LVIEGTGVFV				
								TLAEEVNAAFR				
								VIAWYDNEWGYSQR				
74	gi|115443582	Ribulose bisphosphate carboxylase large chain	21.6/6.41	53.0/6.51	127	10.32	0.03	DTDILAAFR	0.87	0.64	0.89	0.98
								GLTSLDRYKG				
								TFEGPPHGIQVER				
								RVALEACIKIRNQGQN				
Material and Energy metabolite												
1	gi|121083	Glycine dehydrogenase	113.2/6.22	115.4/7.17	136	5.68	0.02	ATLKRLLSEA	0.97	1.38	1.91	1.14
								ASKNKVFKSFIG				
								EVLDYGEFIK KA				
								DETTTLEDVDKLF				
								EYA AFPAAWLRGA				
2	gi|2565305	Glycine decarboxylase P subunit	112.5/6.26	111.9/6.32	73	5.91	0.04	KSFIGMGYYN THIPAVILRNLM	0.94	1.94	1.62	1.31
								GADIAVGSA RFG				
								AEEARKNEMNLRV				
								EYAAFPAAWLRGAK				
3	gi|51090904	Putative glycine dehydrogenase	113.5/6.3	112.4/6.35	162	9.05	0.01	PRRHNSATPAEQ	0.85	4.4	3.32	3.27
								SFIGMGYYNTHVP				
								VCGVLVQYPG TEG				
								IPSSLVRKSPYLTHP				
								HYPVLFRGVNGTV				
								FCDALISIREEIAE				
								EYAAFPAAW LRGA				
25	gi|255567778	Cysteine synthase	55.2/5.33	43.4/7.60	95	10.05	0.02	PSVVCKA	1.33	1.36	3.27	1.36
								KLEIMEPCCS				
								IQGIGAGFVPR				
								YLSSVLFQSIREE				
27	gi|13096165	Chain A, Crystal Structure Of The Complex Between Ferredoxin And Ferredoxin-NADP+ Reductase	53.5/5.43	35.6/7.01	95	14.65	0.03	KKQEEGV	1.15	0.81	3.29	0.65
								DDAPGETW				
								HMVFSTEGKIPYR				
								ERAPENFRVD YAVSREQT				
38	gi|226533016	ATP synthase subunit gamma	60.2/6.3	40.1/8.44	117	14.7	0.03	TTRRRSP	0.96	0.53	0.52	0.36
								SFKRTYRSLD				
								TLLPMSPKGEIC				
								ALQESLASELAAR				
42	gi|343526106	Chromosomal replication initiation protein	50.7/4.22	35.3/6.20	36	13.87	0.02	PGLGKTH	2.28	21.92	80.83	13.17
								LLLIDDIQSL				
								DTLEYLAGQFDS				
								KQDASQMLVIPIDK				
44	gi|75755666	ATP synthase subunit beta	43.6/4.26	53.7/5.17	614	12.45	0.03	GRIVQII	0.97	1.41	1.38	1.28
								NNRVRAVA				
								AHGGVSVGGVGR				
								TATMAYRDVNDVID				
								NIRVAGSVSAG				
								DIIAIGDSDR				
46	gi|150020193	ATP synthase subunit beta	43.1/4.29	51.7/4.92	71	11.09	0.02	FEEGELP	0.91	0.31	0.58	1.42
								AVRTVALD				
								NIAIEHHGFS				
								VALSALTMAEYFR				
								FVQAGSEVSALLGR				
47	gi|50401827	ATP synthase subunit beta	34.5/4.19	53.9/5.17	129	11.65	0.01	TFPPGKL	2.22	4.07	2.06	1.81
								NNRVRAVAMS				
								I FNVLGEPVDN				
								LGPVDSSATFPIHR				
								VVDLLAPYRRGGKIGL				
65	gi|310814906	6-diaminopimelate—D-alanyl-D-alanine ligase	36.3/5.51	50.0/6.75	123	11.72	0.02	FAVTGVS	0.64	1.2	0.91	0.73
								IVDDVLAAL				
								EMLRTVLAAAGR				
								RTPFHMKVAS AGR				
								LPAERAYWLTPEEIL				
Response to stress												
4	gi|357134135	70 kDa heat shock protein	100.2/4.65	73.2/5.04	101	10.81	0.03	VAAMEGGKPTI	1.15	1.21	1.36	1.58
								NEVAEESKQVS				
								VITVPAYFNDSQR				
								EIDEVILVGGSTR				
								DGQTSVEINVLQG				
								TQTIKDALAA LNQ				
5	gi|17737941	Heat shock protein cognate 2	99.3/5.11	70.1/6.76	101	11.06	0.01	DQGNRTTPS	1.07	1.17	2.11	2.02
								DAVVTVPAYF				
								SQRQATKDAGS				
								ARFEELNMDLFR				
								IPCKQQQIFTTYSD				
								RDKCSSEASWLDKN				
6	gi|2501354	Transketolase 7	98.9/5.52	73.9/6.16	131	10.32	0.02	NPYWFNRDRF	1.36	1.4	2.42	1.23
								NEACSLAAHWGL				
								AEGAALESAWNA				
								EHAMGSICNGLA				
								AGAYRAAVQNGE				
								RVSVEAGSTFGW				
11	gi|50897038	Methionine synthase	103.6/6.24	84.8/5.68	201	7.45	0.04	YLFAGVVDGR	0.79	1.13	0.23	0.03
								IQEELDIDVLVHGEPER				
								VDAGGIVIIDAAR				
								GVVYGAGIGGVYDIHSR				
18	gi|115448091	Os02g0698000	75.6/5.07	45.2/5.68	98	12.9	0.01	CTTNTSFRQ	1.15	5.41	15.08	3.67
								FGGAAEPPKG				
								LLDPPELIQPPK				
								NFNPVYLFDE				
								GSSITWVPCGR				
40	gi|162461348	Isoflavone reductase homolog IRL	50.4/6.26	32.8/5.69	144	18.77	0.02	VGGTGYLG	0.95	0.35	0.25	0.18
								LVSAVKGADV				
								FFPSEFGLDVDR				
								TLSHNELLSLWEK				
								DPAKGVDASELYPDV				
62	gi|357121842	29 kDa ribonucleoprotein	34.5/5.34	29.4/4.79	87	12.36	0.03	VCGPVRAA	1.03	1.26	1.46	1.64
								TGRSRGFGFVT				
								VNSGPPPPRDEFAPR				
63	gi|357136935	Dehydrin COR410-like	30.5/5.23	5.2/27.50	179	16.14	0.01	KDKKVVT	2.64	5.71	7.86	8.88
								KGKKIKKGH				
								KTAVAKKGKIK				
								AATVSSKKGIGKIM				
69	gI:81362226	9-cis-epoxycarotenoid dioxygenase 2	56.9/6.78	43.3/9.11	160	8.61	0.03	SSVTSAR	1.45	2.55	3.35	3.63
								TDRIINGVY				
								DGDTVGRYDD				
								RRIASVNVGMVN				
								VHDAAGTSVVNAA				
Ion binding												
7	gi|66806687	Arginyl-tRNA synthetase	98.7/5.59	85.3/6.36	148	5.01	0.03	IYLLNIK	0.86	0.99	1.7	1.69
								ALTKEEIEK				
								EYLTTILKLW				
								MGFVDISDDGR				
22	gi|85701941	Tripartite motif-containing protein 75	63.4/5.49	54.5/7.53	94	7.49	0.04	TDPVTVE	1.09	0.98	3.9	3.14
								HGRHQVLS				
								RLLDNIAAL				
								EHHGSSLRDLL				
24	gi|357147655	Quinone oxidoreductase-like protein At1g23740	57.8/5.39	39.8/8.29	105	11.46	0.02	LSSVSGA	0.62	0.58	1.41	1.42
								EGGSVVV				
								LTGAVTPPGFR				
								GPFSFPQVVE				
								AFSYLE TGR				
26	gi|348504930	Prolyl 3-hydroxylase 2-like	52.4/5.31	77.6/5.56	173	4.82	0.04	SAGVL	1.94	1.77	5.13	3.79
								QDFNRRI				
								AEFTEGILST				
								TAIKGQQDHR				
52	gi|357200814	ATPase P	38.5/4.83	87.9/5.21	76	4.2	0.04	AACASR	2.72	2.25	2.23	0.71
								VLSFPLL				
								ELVEPTDF				
								EGIAVGELV				
								EQHMHGLENAGK				
56	gi|20939	Chlorophyll a/b-binding protein	36.1/5.05	28.8/5.31	83	13.16	0.02	RITMRR	0.85	0.38	0.3	0.26
								ELEVIHCR				
								LIEGYRVGGG				
								QAIVTGKGPIE				
59	gi|131384	Oxygen-evolving enhancer protein 1	46.3/5.15	35.1/6.25	95	9.74	0.03	AFGLEHY	11.05	6.93	17.33	6.39
								TSALVVSGA				
								DGI DYAAVTVQLPGGER				
64	gi|131394	Zinc finger CCCH domain-containing protein 56	35.7/5.56	27.42/8.84	155	12.02	0.03	PAVGRT	1.23	1.24	3.39	3.45
								NTDFVA				
								YSGEGFK				
								QQSYGGKTDSEG				
Antioxidant activity												
49	gi|195626524	2-cys peroxiredoxin BAS1	33.7/4.68	28.3/5.81	149	18.46	0.02	APDFEAEA	1.12	1.28	1.68	1.83
								VFDQEFINVK				
								AFGVLIPDQGIALR				
								EGVIQHSTINNLAIGR				
50	gi|195626524	2-cys peroxiredoxin BAS1	33.8/4.74	28.3/5.81	112	10.38	0.02	APTPAA	1.52	2.03	4.28	2.78
								RYEEFEK				
								AFGVLIPDQGIALR				
57	gi|559005	Ascorbate peroxidase	38.9/5.18	27.7/5.43	142	16.4	0.01	RGLIAEK	1.42	1.09	0.98	1.19
								IKEQFPI LS				
								LPDATKGSDHLR				
								EKYAADEDAFFAD				
80	gi|42408425	Superoxide dismutase	22.4/5.14	21.4/5.79	103	19.43	0.01	AMAAQ	1.24	1.32	1.82	2.28
								AAGPAGAA				
								AFVVHELED				
								DLGKGGHEL				
								SLSTGNAGGR				
Signal transducer activity												
29	gi|213958273	Putative mitochondrial cysteine synthase precursor	54.4/5.49	22.6/5.36	190	22.43	0.01	SVLVEATSGN	2.21	7.81	1.04	1.5
								TGIGLAFIAASR				
								VDIFIGGIGTGGT				
								IQGIGAGFVPRNL				
43	gi|12081917	Cytosolic cysteine synthase	49.9/4.26	34.4/5.93	129	14.46	0.02	IAKDVT	0.71	0.56	1.03	1.5
								VLIEPTSGN				
								LTDPAKGMK				
								PGPHKIQGIGA				
								LIVVIFPSFGER				
53	gi|162462421	Adipokinetic hormone 1 precursor	36.7/4.89	7.5/8.55	120	25.76	0.01	CFIMAEA	1.58	1.43	0.91	0.88
								AAIAGTVSCR				
72	gi|328885213	Aspartyl/glutamyl-tRNA(Asn/Gln) amidotransferase subunit C	8.6/6.80	10.7/4.72	98	15.31	0.02	LARLELK	0.92	1.02	1.85	1.9
								VPQILGED				
Cell structure												
51	gi|264677437	Long-chain-fatty-acid—CoA ligase	36.0/4.73	59.9/5.80	109	6.09	0.03	ADIFE	2.62	0.98	0.73	0.54
								FFACSKIG				
								CTGGTTGLPK				
								GHMPLGYYGDPRK				
Transport												
21	gi|357149925	Elongation factor Tu	68.5/5.49	50.6/5.88	72	9.34	0.03	PSTSSSK	1.36	2.66	4.3	3.87
								NIGTIGH				
								DQVDDEEL				
								LELVDLEVR				
								TPHTKFEAVVYVL				
60	gi|31211267	ShnG	38.5/5.33	19.6/9.61	116	21.67	0.01	PAKESK	1.27	3.52	1.81	2.36
								RQQVARRTD				
								DAGFFGISPRE				
								VSLVSPIRTGLPP				
76	gi|341616065	Two-component system sensor histidine kinase	17.3/4.37	41.2/5.28	68	8	0.04	VHRL	1.07	1.36	3.8	1.43
								AEARAS				
								QIARGHAG				
								SLELAGACPTVFR				
Predicted protein												
20	gi|170782258	Putative hydrolase	68.8/5.38	36.3/4.57	91	14.29	0.02	REGVD	1.04	3.58	7.19	5.04
								RDGLHR				
								GTPVVAAVLT				
								DRLLLGANAMWG				
								SVMVGFLARVSATSGPA				
30	gi|326506704	Predicted protein	53.4/5.59	41.1/6.08	143	11.83	0.02	SVLVEATSG	1.01	1.02	2	2.06
								NTGIGLAFIAASR				
								LIVVVFPSFGERY				
								LSSVLFQSIRE				
58	gi|14091862	Putative hydrolase	41.6/5.22	41.4/9.17	196	9.65	0.03	NETFAER	0.8	0.14	1.52	1.79
								AVSAIVSCLLGPDR				
								ITIFAGDVVPRKKPD				
61	gi|14091862	Putative hydrolase	40.3/5.41	41.4/9.17	132	9.92	0.04	ISFNETFAER	1.37	2	3.75	1.43
								AVSAIVSCLLGPDR				
								ITIFAGDVVPRKKPD				

a. Database accession numbers according to NCBInr.

b. Experimental Mw/pI.

c. Theoretical Mw/pI.

d. Mascot search score against the database of NCBInr.

e. Sequence coverage.

f. Protein spots showing significant change in abundance (>1.5-fold) compared with control, as determined by LSD test (*p*<0.05).

### Effect of drought stress on antioxidant enzyme activities

**Figure 4:**
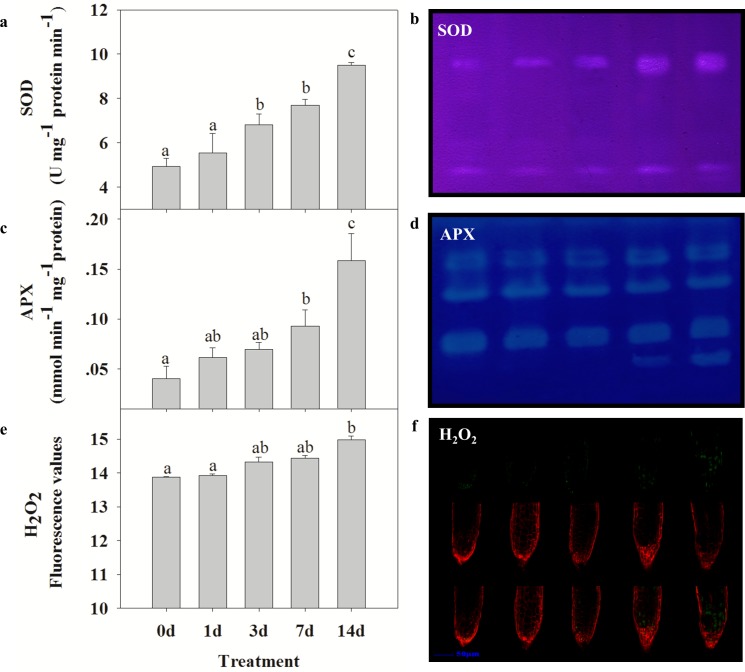
Effects of drought stress on antioxidant enzymes and H_2_O_2_ accumulation in *S*. *purpurea* seedlings. (A, C) Effects of different RWCs on SOD and APX activities in *S*. *purpurea* seedlings, as determined by a colorimetric method. (B, D) Effects of drought on SOD and APX activities as determined by activity staining. (E) H_2_O_2_ formation, as visualized by infiltrating H2DCF-DA into roots of *S*. *purpurea* and observing fluorescence. (F) Average fluorescence intensity of H_2_O_2_. Data are mean values ± SE obtained from three independent experiments (*n* = 30). Different letters within a column indicate significant differences (*p*<0.05; one-way analysis of variance and Tukey’s test.). Bar = 50 μm.

The results of the 2-DE and MS analyses indicated that antioxidant enzymes accumulated in seedlings of *S*. *purpurea* under drought stress. This finding was expected, because drought stress is known to induce increases in the activities of antioxidant enzymes such as SOD, APX, and CAT, which increase the capacity to eliminate ROS, in various plant species [33]. The highest concentrations of SOD and APX were observed on days 14 and 3 of the drought treatment, respectively. The abundance of these proteins increased by 2.28-fold (SOD; Fig. 2A, spot 80) and 1.42-fold (APX; Fig. 2A, spot 57), compared with their respective levels on day 0. There was also an increase in the abundance of 2-cys peroxiredoxin BAS1 (Fig. 2A, spots 49 and 50; 1.83-fold on day 7, 4.28-fold on day 14), suggesting that this protein also contributed to the antioxidant system in *S*. *purpurea*. To confirm these results, we measured the activities of antioxidant enzymes in the drought-stressed seedlings. There were significant increases in SOD and APX activities as the RWC decreased during the drought treatment (Fig. 4A and C). Similar trends were observed in the native gel activity assays of drought-stressed *S*. *purpurea* seedlings (Fig. 4B and D).

### Drought stress induced the accumulation of ROS

To investigate the interaction between antioxidant proteins and ROS in drought-treated *S*. *purpurea* seedlings, we quantified H_2_O_2_ production in the root tips. Compared with the control, the drought-stressed plants showed a slow increase in ROS production under prolonged drought stress, as revealed by the increase in H2DCF-DA fluorescence as the drought treatment continued (Fig. 4E and F). The level of H_2_O_2_ peaked when the RWC decreased to approximately 5%. As shown in Fig. 4F, weak fluorescence (green) was emitted from the driest sample (14 d of drought treatment). These results strongly indicated that enhanced antioxidant enzyme activities allowed the drought-stressed plants to maintain their cellular redox status at an acceptable level to avoid damage from excess ROS.

### Characterization of carbonylated proteins in *S*. *purpurea* under drought stress

**Figure 5:**
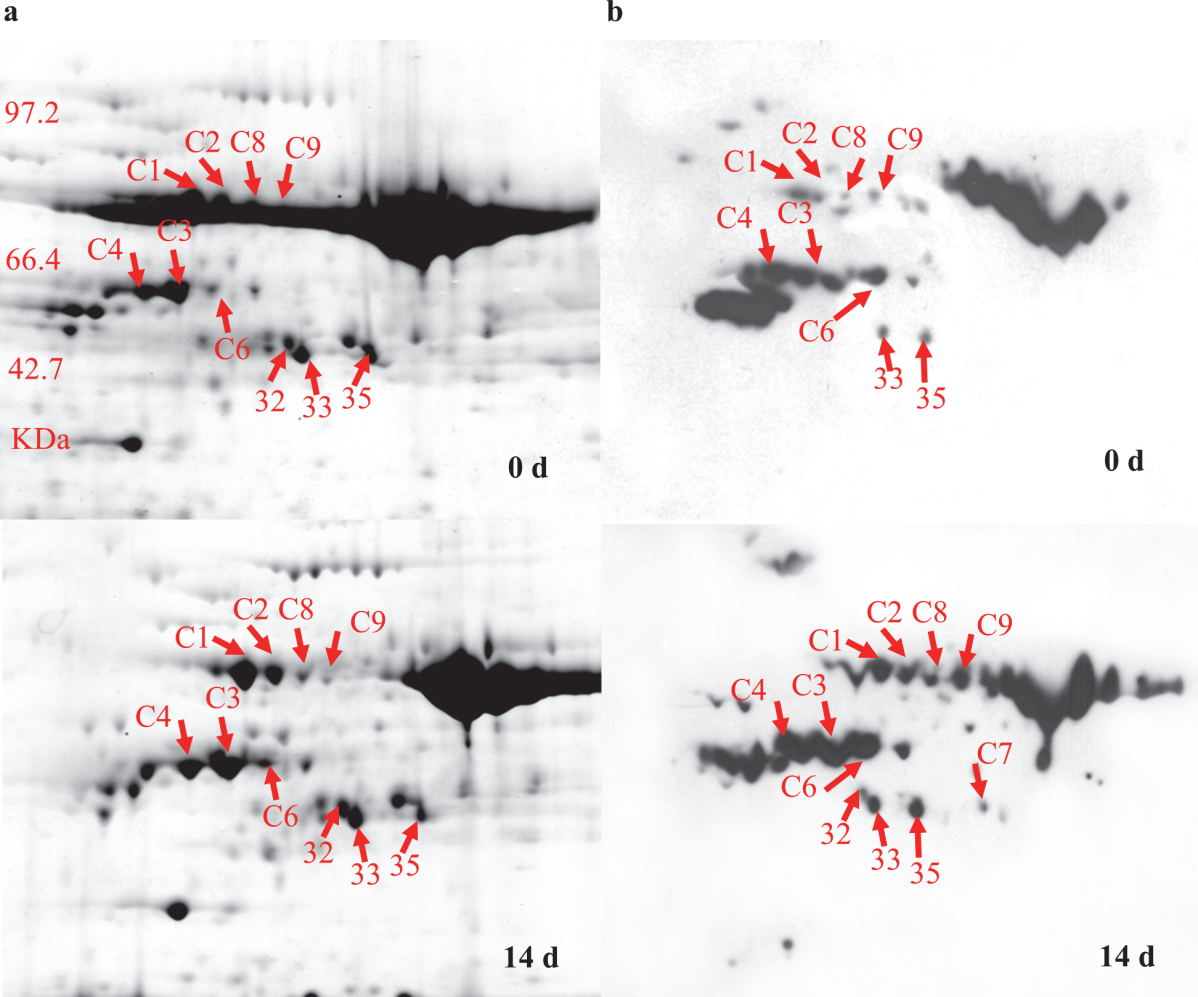
Detection of carbonylated proteins of *S*. *purpurea* seedlings. Carbonylated proteins were analyzed by 2D-PAGE. (A) Gels stained to detect proteins. (B) Anti-DNP immunoassays of gels to detect carbonylated proteins. Labeled proteins are listed in Table 2.

To further characterize the oxidative stress induced by drought stress in *S*. *purpurea*, we used a proteomic immunochemical approach to identify carbonylated (oxidized) proteins. Proteins were extracted from leaves of plants in the control (0 d) and from those subjected to 14 d of drought, and then separated by 2-DE. The carbonylated proteins were cut from 2D gels, digested with trypsin, and then identified by MS as described above. The identified protein spots are shown in enlarged images of 2D gels (Fig. 5A and B) and further details are provided in Table 2. The carbonylated proteins showing increased abundance under drought stress included RuBisCO activase (spots C3, C4, C7, C8 and 32), ATP synthase beta subunit (spots C1 and C2), phosphoglucan, water dikinase (spot C5), oxygen-evolving enhancer protein 1 (spot C6), 2-phosphoglycerate dehydratase 1 (spot C9), chloroplastic aldolase (spot 33), and fructose 1, 6-bisphosphate aldolase precursor (spot 35). In another study, the abundance of oxidized proteins increased after exposure to different biotic and abiotic stresses [26]. Our results showed that proteins in the chloroplasts, plastids, mitochondria, and cytosol were oxidized under drought stress in *S*. *purpurea*.

**Table 2:** Identification of oxidation-sensitive differentially expressed proteins in drought-stressed *S*. *purpurea* leaves.

Spot^a^	Protein description	Exp. Mw/pI^b^	Theo. Mw/pI^c^	Acc. No.^d^	Score	Species
C1	ATP synthase beta subunit	69.97/5.41	52.99/5.17	gi110915694	1,080	Festuca arundinacea
C2	ATP synthase beta subunit	69.78/5.51	52.98/5.11	gi110915596	969	Brachypodium pinnatum
C3	Ribulose-1,5-bisphosphate carboxylase activase	59.76/5.37	21.74/4.78	gi13569643	230	Oryza sativa Japonica Group
C4	RuBisCO activase small isoform precursor	58.65/5.20	48.13/5.85	gi62733169	380	Oryza sativa Japonica Group
C6	Oxygen-evolving enhancer protein 1	60.16/5.57	52.68/8.70	gi514777415	515	Setaria italica
C7	Ribulose bisphosphate carboxylase/oxygenase activase	57.67/6.05	30.60/5.10	gi149392725	367	Oryza sativa Indica Group
C8	Ribulose-1,5-bisphosphate carboxylase/oxygenase	69.94/5.62	49.92/6.23	gi2465451	101	Anomochloa marantoidea
C9	2-phosphoglycerate dehydratase 1	69.68/5.76	48.26/5.20	gi119355	115	Zea mays

a. Spot number in 2-DE gel as shown in Fig. 2A.

b. Experimental molecular weight and pI.

c. Theoretical molecular weight and pI.

d. Accession number in NCBI database.

### Dynamic changes in levels of osmolytes, ABA, and RuBisCO in *S*. *purpurea* under drought stress

**Figure 6:**
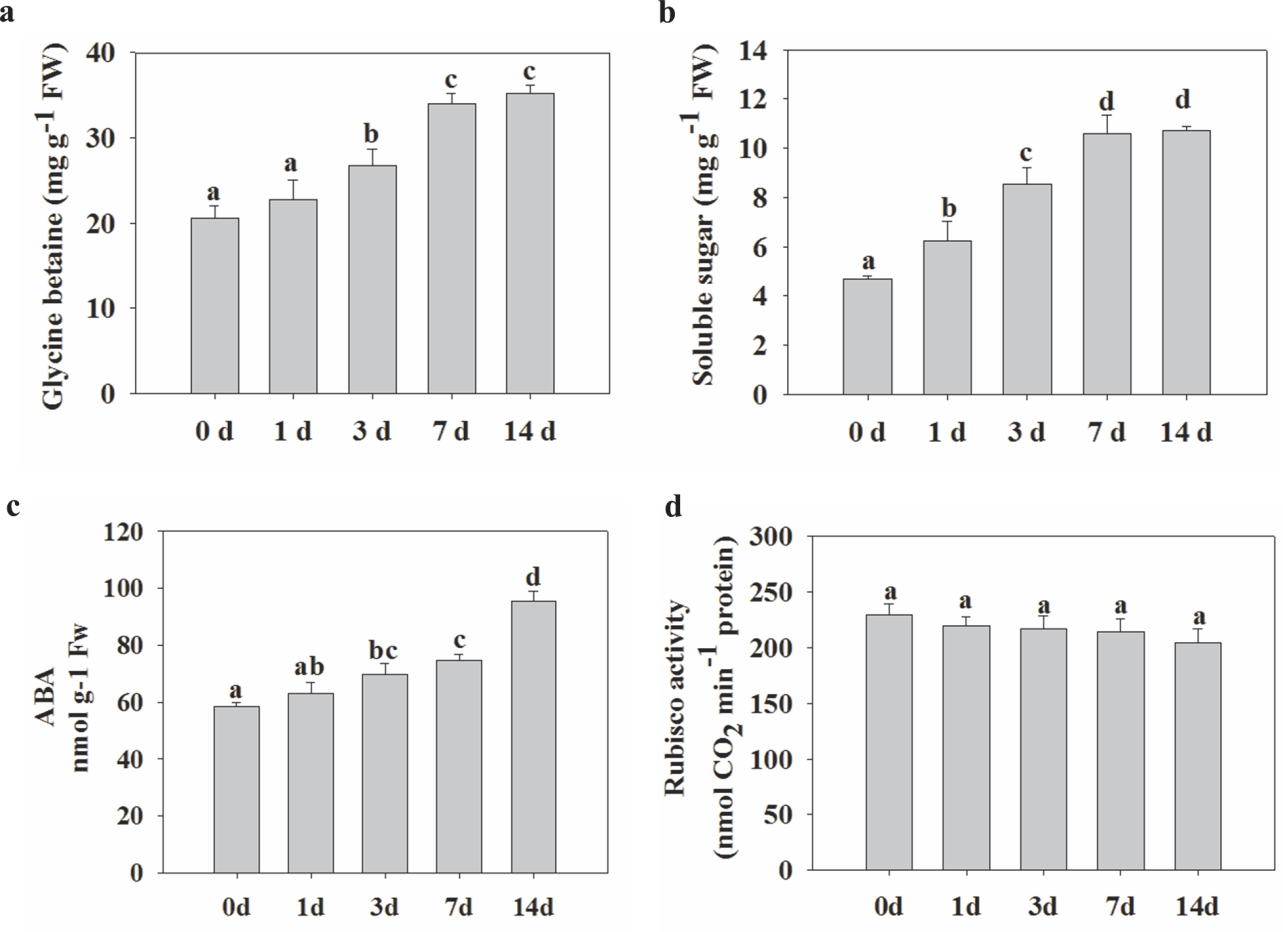
Effects of drought on glycine betaine, soluble sugars, and ABA contents and RuBisCO activity in *S*. *purpurea* seedlings.

Osmolytic adjustment is a vital mechanism in the tolerance of plants to dry environments [34], and glycine betaine and soluble sugars are important metabolites in the drought stress response. The proteomic analyses showed that several osmolyte proteins responsible for the synthesis of glycine betaine and soluble sugars were upregulated under drought stress (Fig. 2A, spots 1, 2, and 65). The concentrations of glycine betaine and soluble sugars were significantly higher (*p*<0.05) in drought-stressed seedlings than in control seedlings after 14 d of drought treatment (Fig. 6A and B). The ABA content in *S*. *purpurea* seedlings gradually increased under prolonged drought stress, reaching a maximum at day 14 (Fig. 6C). There were no significant differences in RuBisCO activity between drought-stressed plants and controls (Fig. 6D).

### Western blot analyses

The proteomic analysis revealed that levels of ABA signaling-related proteins increased in response to drought stress. The plant hormone ABA, which is synthesized by NCED2, modulates tolerance to drought stress [8]. Our proteomic profile showed that NECD2 was upregulated under drought stress. This was validated by the western blot analysis. The increased abundance of several defense proteins [dehydrin COR410-like protein (DHN) and heat shock protein 70 (HSP 70)] was also confirmed by western blot analyses.

## Discussion

### Changes in growth and photosynthesis-related proteins under drought stress

Photosynthesis is an important metabolic process that plays a central role in regulating plant responses to drought stress. It is mainly regulated via photochemical reactions related to energy production, gas exchange, and CO_2_ fixation and assimilation. Some of the proteins involved in photochemical pathways, such as ATP synthase family proteins (Fig. 2A, spots 47, 46, 47, and 52), showed increased abundance in *S*. *purpurea* under drought stress, suggesting that they were involved in providing adequate and affordable energy. Increased ATPase activity may also function in homeostasis, to maintain the cytoplasmic pH near neutral. The 1.28- to 4.07-fold increases in the abundance of ATP synthase family proteins under drought stress was closely correlated with increased abundance of RuBisCO activase (Fig. 2A spots 14, 16, and 70), which is involved in regenerating carbamylated active sites in RuBisCO to maintain its activity [35]. In another study, the RuBisCO transcript level in pea increased two-fold when the soil RWC decreased by 50% [36].

Phosphoribulokinase (PRK) is involved in regenerating RuBisCO during the carbon assimilation process [37]. In the present study, the increased abundance of PRK (Fig. 2A, spot 17) (1.62- to 4.17-fold) during days 1–7 of drought stress suggested that RuBisCO regeneration was promoted under drought stress. A set of proteins, including PRK, that showed 1.3- to 5.0-fold increases under water stress were identified in maize [38]. The accumulation of RuBisCO activase in *S*. *purpurea* under drought stress suggested that the photosynthetic CO_2_ fixation capability was enhanced via increased RuBisCO activity. RuBisCO for carboxylation and glyceraldehyde 3-phosphate dehydrogenase (GADPH) for carbon reduction were identified as key enzymes for maintaining higher photosynthetic capacity in drought-tolerant compared with drought-sensitive *Kentucky bluegra*ss under drought stress [30]. In the present study, GADPH A (Fig. 2A, spot 73) and GADPH B (Fig. 2A, spot 23) were differentially regulated in the leaves of *S*. *purpurea* under drought conditions. Together, these results indicated that a higher photorespiration rate contributed to drought tolerance in *S*. *purpurea*.

Our proteomic analysis showed that drought stress resulted in oxidative damage to proteins (Fig. 5B) in *S*. *purpurea* seedlings. This was revealed by the increased abundance of carbonylated proteins (i.e., oxidized proteins) in drought-stressed plants, as detected in the DNP immunoassay. One of the oxidation-sensitive proteins in *S*. *purpurea* seedlings was RuBisCO (Fig. 5, spots C3, C8, C4, C7 and 32). Photosynthetic efficiency can be enhanced by increased carboxylase activity and/or by decreased oxygenase activity of RuBisCO [39]. Carbonylation of RuBisCO subunits resulted in decreased photosynthesis in pea under cadmium stress [40]. In the present study, the main carbonylated proteins were RuBisCO (Fig. 5, spots C3, C8, C4, C7, and 32). However, some RuBisCO proteins were not oxidized (Fig. 2, spots 14, 16, and 70) and were expressed at high levels under drought stress. There were no significant differences in RuBisCO activity in *S*. *purpurea* seedlings between drought-stressed and control plants. This result suggested that drought stress might inhibit photosynthesis via oxidation of RuBisCO, and that a compensatory upregulation of unoxidized RuBisCO proteins occurred during the response of *S*. *purpurea* seedlings to drought.

### Production of ROS, carbonylation of proteins, and antioxidant capacity of *S*. *purpurea* under drought conditions

The regulation of ROS that accumulate under biotic or abiotic stress is an important factor in stress responses, because excess ROS can severely disrupt metabolic processes and damage various cellular components, including nucleic acids, lipids, and proteins. Plants have evolved ROS-scavenging enzymes including SOD, GR, APX, CAT, GPX, and peroxiredoxin, which provide cells with an efficient mechanism to neutralize ROS. Drought stress has been shown to activate genes encoding antioxidant enzymes such as peroxidase, SOD, and APX in *Alfalfa* and various other plant species [41]. In our proteomic analysis, we detected differential expression of antioxidant proteins; 2-Cys peroxiredoxin BAS1, APX 1, and SOD (Fig. 2A). 2-Cys peroxiredoxin BAS1 reduces hydrogen peroxide and alkyl hydroperoxides using reducing equivalents produced in the thioredoxin or glutaredoxin system. This protein was shown to be upregulated by drought-induced oxidative stress in rice chloroplasts, and it was able to decrease H_2_O_2_ levels [42]. It has also been shown to function as an electron acceptor in CDSP32-driven electron-transfer, thereby protecting the photosynthetic apparatus from oxidative damage in *Arabidopsis* [43]. Our proteomic results show that the abundance of 2-Cys peroxiredoxin BAS1 in *S*. *purpurea* increased as the RWC decreased (Fig. 2A, spots 49 and 50).

Our results showed that the greatest abundance of APX1 and SOD was in *S*. *purpurea* seedlings in the driest soil (approximately 5% water content) (Fig. 2A, spots 57 and 80). In plants, SOD and APX are two of the main ROS-scavenging enzymes, and constitute a highly efficient system for removing superoxide and hydrogen peroxide. Hydrogen peroxide is converted to H_2_O by APX, which uses ascorbate as the substrate, and SOD catalyzes the dismutation of O_2_^−^ into O_2_ and H_2_O_2_ [44]. To further investigate the relationship between H_2_O_2_ and antioxidant enzymes, we measured the activities of APX and SOD, and quantified H_2_O_2_ production in drought-stressed *S*. *purpurea*. The activities of SOD and APX increased during acclimation to drought (Fig. 4). In another study, SOD activity in the leaves of *Capparis ovata* was 1.7-fold and 1.8-fold higher on days 7 and 14 of drought stress, respectively, compared with their levels in the control; these increased activities prevented ROS damage under drought stress [45]. The results of the enzyme activity assays and proteomics analysis were consistent in this study. Together, our results indicated that during the adaptive response of *S*. *purpurea* seedlings, drought induced the expression of antioxidant proteins that neutralized excess ROS and minimized damage.

The degree of protein oxidation appears to be related to the amount of ROS generated and the balance between the rate of protein oxidation and the rate of protein degradation under stress conditions. We observed that *S*. *purpurea* could endure oxidative damage caused by decreases in RWC to approximately 5% (Fig. 1A). Another antioxidant defense system, the NO-mediated S-nitrosylation of critical protein thiols, can prevent protein oxidation in plants. We identified several homologs of S-nitrosylation-sensitive proteins that have been reported in *Arabidopsis* [46]. The abundance of methionine synthase (Fig. 2A, spot 11) and elongation factor Tu (Fig. 2A, spot 21) increased to different degrees in drought-stressed *S*. *purpurea*. However, further research is required to determine whether S-nitrosylation-sensitive proteins are S-nitrosylated, and whether NO provides antioxidant protection to cells by S-nitrosylation of functional proteins in the adaptive response of *S*. *purpurea* to drought conditions.

### Expression patterns of stress-responsive proteins in *S*. *purpurea*

**Figure 7:**
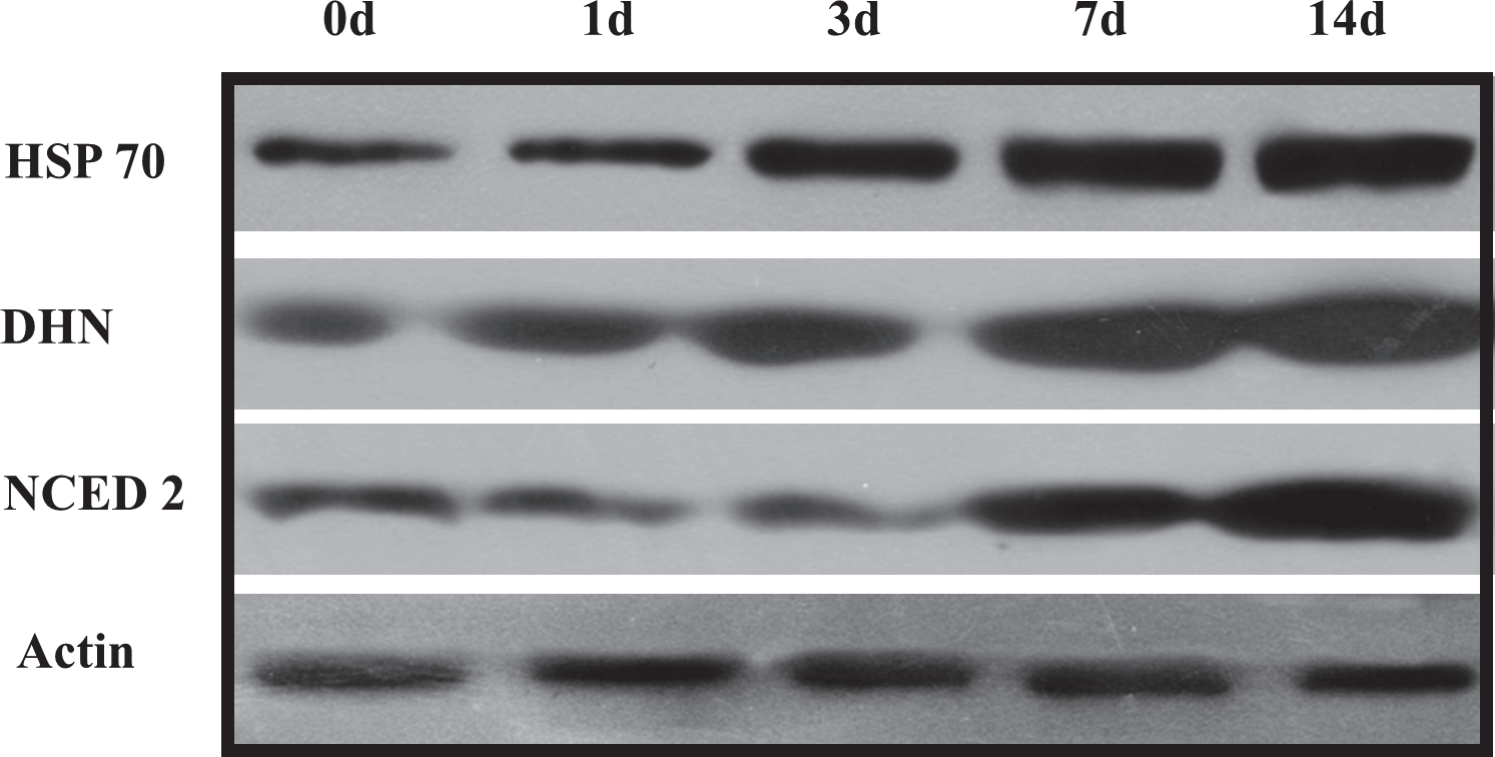
Western blotting analysis of proteins from *S*. *purpurea* seedlings. Total protein samples were separated by SDS-PAGE and electroblotted onto polyvinylidene difluoride membranes. Membranes were probed with anti-HSP, anti-NCED2, and anti-dehydrin primary antisera. Actin served as the protein loading control.

Dehydrin proteins, which are members of the LEA protein family, were named for their proposed function in plants under drought stress. Accumulation of dehydrin proteins is associated with drought responses in many plant species including *Arabidopsis*, rice, wheat, and poplar [10, 35, 42, 47]. Accumulation of members of the LEA protein family, including dehydrins, is often upregulated (>2-fold) in the stress responses of plants [47]. The results of our 2-DE analysis showed that a dehydrin (Fig. 2A, spot 63) was strongly expressed in leaves of *S*. *purpurea* when the RWC decreased from approximately 80% to 5%. Similar results were observed in the western blot analysis. Together, these results indicate that accumulation of dehydrin proteins may have enhanced drought tolerance by protecting cells against dehydration (Fig. 7). Dehydrins may play an important role in *S*. *purpurea* by increasing the concentrations of cellular solutes during acclimation to drought conditions.

Heat shock protein 70 (HSP70) (Fig. 2A, spot 4) and heat shock protein cognate 2 (Hsc70–2, spot 5) showed increased abundance in leaves of *S*. *purpurea* under drought stress. The functions of Hsc70–2 and HSP70 are similar; both prevent protein aggregation, assist non-native proteins to refold, and maintain organellar precursor proteins under stress conditions. The expression of HSP70 has been reported to increase in many plant species under stress conditions; for example, by more than 8-fold in drought-stressed *Arabidopsis*, and by almost 10-fold in drought/heat stressed wheat, as compared with controls [48, 49]. One example of the function of HSP is to control the correct folding of RuBisCO. A more efficient RuBisCO could reduce photosynthetic water use, thereby increasing tolerance to drought stress [50]. Our results showed that RuBisCO was an oxidation-sensitive protein (Fig. 5B, spot 32, 34). Together, these results suggested that drought-induced HSP70 could help to ameliorate oxidative damage to RuBisCO by ensuring its correct folding to maintain the photosynthetic capability of *S*. *purpurea* leaves.

### ABA biosynthesis-related protein changes during drought stress

In plants, ABA rapidly accumulates in response to various stresses, including drought and high salinity, and mediates diverse resistance mechanisms. Our 2-DE results showed that the key ABA catabolism enzyme NCED2 (Fig. 2A, spot 69) was expressed most strongly in plants in the driest soil. Similar results have been reported for a number of plant species including *Arabidopsis*, rice, and barley [51]. The results of the western blot analysis confirmed that the abundance of NCED2 increased along the drought-stress gradient (Fig. 7). In plants, ABA plays a central role in stomatal closure during drought stress [52]. The ABA signal in guard cells results in the production of nitric oxide and H_2_O_2_, leading to stomatal closure [53]. Stomatal closure is a protective mechanism to avoid excessive water loss from leaves. Our study demonstrated that accumulation of ABA induced stomatal closure, resulting in reduced stomatal conductance (Fig. 1B). These results suggested that ABA is produced by *S*. *purpurea* seedlings under drought conditions, reducing stomatal conductance and leading to relatively stable photosynthesis in the leaves.

### Expression patterns of material- and energy metabolite-related proteins in *S*. *purpurea*

Osmotic adjustment is a key adaptive response to drought stress in many plants and other organisms. This process can provide resistance to drought, within species-specific limits, by maintaining turgor and cell volume [4]. The most important osmolytes in plants are glycine betaine and soluble sugars. Accumulation of these substances in plants was shown to enhance the ability of thylakoid membranes to maintain photosynthetic efficiency under drought stress [54]. We detected increases in the amounts of glycine betaine and soluble sugars in *S*. *purpurea* seedlings under drought stress. The higher glycine betaine and soluble sugars concentrations in the drought-stressed plants were accompanied by increased abundance of proteins involved in the biosynthesis of glycine betaine and soluble sugars (Fig. 2A, spots 1, 2, and 65). Thus, glycine betaine and soluble sugars appear to play important roles in osmoregulatory drought resistance of *S*. *purpurea*. Similarly, drought stress was reported to increase the levels of glycine betaine and soluble sugars in wheat, thereby improving drought resistance and maintaining photosynthetic activity and metabolism [55].

## Conclusion

**Figure 8:**
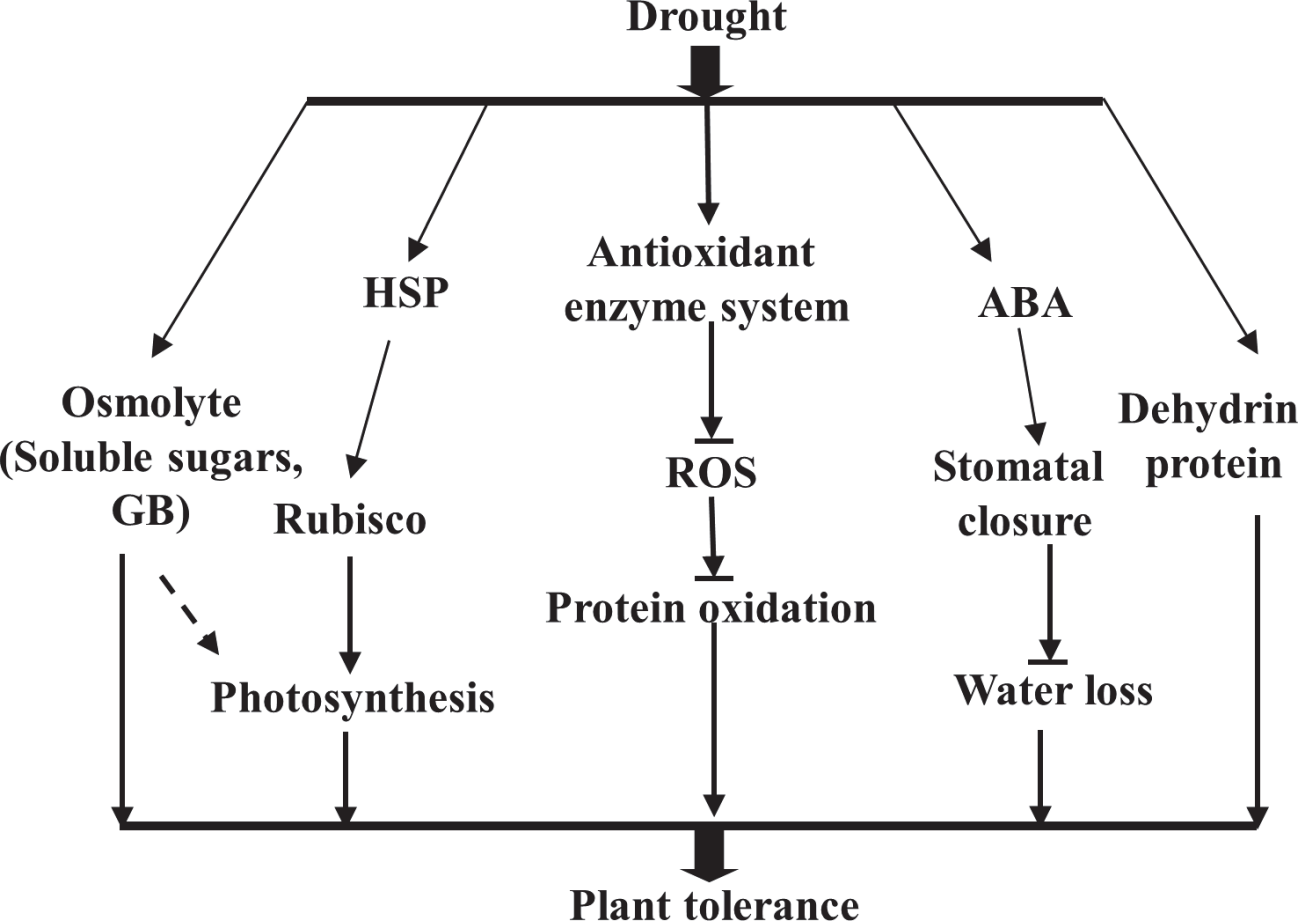
Proposed model of multiple strategies of *S*. *purpurea* to adapt to drought conditions.

The responses of the stress-resistant forage species *S*. *purpurea* to drought are complex. Drought conditions led to the accumulation of compatible solutes (glycine betaine, soluble sugars) and proteins (PRK and HSPs), which allowed the plant to maintain its photosynthetic capacity under drought stress. There were also substantial increases in the abundance of antioxidant enzymes to scavenge toxic ROS, and changes in the expression of numerous other proteins, including dehydrins. These changes enhanced stress tolerance via ABA-mediated regulation of stomatal conductance to reduce water loss (Fig. 8). All of these responses may help to alleviate the negative effects of drought by preventing damage to vital cell constituents and enhancing osmoregulation.
